# Conductive Polymer Combined Silk Fiber Bundle for Bioelectrical Signal Recording

**DOI:** 10.1371/journal.pone.0033689

**Published:** 2012-04-06

**Authors:** Shingo Tsukada, Hiroshi Nakashima, Keiichi Torimitsu

**Affiliations:** Materials Science Laboratory, NTT Basic Research Laboratories, NTT Corporation, Atsugi, Kanagawa, Japan; Indiana University School of Medicine, United States of America

## Abstract

Electrode materials for recording biomedical signals, such as electrocardiography (ECG), electroencephalography (EEG) and evoked potentials data, are expected to be soft, hydrophilic and electroconductive to minimize the stress imposed on living tissue, especially during long-term monitoring. We have developed and characterized string-shaped electrodes made from conductive polymer with silk fiber bundles (thread), which offer a new biocompatible stress free interface with living tissue in both wet and dry conditions.

An electroconductive polyelectrolyte, poly(3,4-ethylenedioxythiophene) -poly(styrenesulfonate) (PEDOT-PSS) was electrochemically combined with silk thread made from natural *Bombyx mori*. The polymer composite 280 µm thread exhibited a conductivity of 0.00117 S/cm (which corresponds to a DC resistance of 2.62 Mohm/cm). The addition of glycerol to the PEDOT-PSS silk thread improved the conductivity to 0.102 S/cm (20.6 kohm/cm). The wettability of PEDOT-PSS was controlled with glycerol, which improved its durability in water and washing cycles. The glycerol treated PEDOT-PSS silk thread showed a tensile strength of 1000 cN in both wet and dry states. Without using any electrolytes, pastes or solutions, the thread directly collects electrical signals from living tissue and transmits them through metal cables. ECG, EEG, and sensory evoked potential (SEP) signals were recorded from experimental animals by using this thread placed on the skin. PEDOT-PSS silk glycerol composite thread offers a new class of biocompatible electrodes in the field of biomedical and health promotion that does not induce stress in the subjects.

## Introduction

Electrode materials for recording biomedical signals are expected to be soft, hydrophilic and electroconductive to make them suitable for water rich environments and the functionalities of living tissue. Metal electrodes have been widely used for bio electrodes [1]. Conventional metal electrodes are hydrophobic and made of hard material. The interfacial mismatch between living tissue and engineered electrodes causes an increment in the contact resistance and noxious stimulation induced by the mechanical strain between the electrode and tissue [1], [2]. Although gel or paste electrolytes are usually used to improve the interfacial mismatch between metal electrodes and living tissues, they disperse easily with time, and this results in an increase in both contact resistance and mechanical strain.

Poly(3,4-ethylenedioxythiophene) poly(styrenesulfonate) (PEDOT-PSS) has been widely used for electrodes in electroluminescent displays or for antistatic coating [3]–[7]. PEDOT-PSS has been examined as a bioelectrode material because of its functionalities; its highly electroconductive, hydrophilic and biocompatible characteristics meet the requirements for biomedical electrode materials [8].

However, in wet conditions, PEDOT-PSS has a hydrogel property, which reduces its mechanical strength according to its water content [4]. The result is exfoliation from the substrates and a loss of electrical conductivity. The repetition of wet and dry cycles accelerates the disruption of PEDOT-PSS. For biomedical use, PEDOT-PSS must be improved so that it is more durable when it becomes wet with sweat or fluid originating from living tissue.

For the fabrication of biomedical electrodes, such as skin surface electrodes for electrocardiography (ECG), it must be possible to form PEDOT-PSS, whose use has thus far been limited to micro scale films or fibers [4], [9], on a millimeter scale to obtain stable recordings from high impedance skin surfaces.

Dry electrodes have been developed for medical, sports and ubiquitous healthcare systems without using electrolyte pastes for application to long-term monitoring or unobtrusive sensing systems. E-textile bioelectrodes or wearable electrodes commonly use textiles that are woven or stitched with metal coated fibers or fine metal wires to conduct body signals from the skin surface [10]. Synthetic fibers including nylon and polyester are coated with silver or other metals to provide electrical conduction [11]. Silver plated nylon yarns have been frequently used for e-textile bioelectrodes especially in the field of sports.

Silk fiber, produced by the silkworm *Bombyx mori*, is one of the most widely used natural fibers for textile [12]. Moreover, it has been used in the biomedical field as, for example, suture thread or as the base for an ointment, by virtue of its excellent strength and biocompatibility. Genetically modified silk fibroin proteins as well as commercially available silk fibers have been used to design functional material products including scaffolds for cell culture in tissue engineering, biocompatible electrodes and biological glue [13],[14]. Recently, it was reported that electroconductive silk fiber had been fabricated by staining PEDOT-S (poly(4-(2,3-dihydrothieno [3,4-b] [1], [4]dioxin-2yl-methoxy)-1-butanesulfonic acid)) for use in fabrics and fiber transistors [15], [16].

In this paper, we present a microfiber reinforced composite material made of PEDOT-PSS and natural silk fiber bundles (thread) from *Bombyx mori* for use as a biomedical electrode that is water resistant, electroconductive and flexible. PEDOT-PSS was electrochemically deposited into the silk thread to form a string-like fiber composite material that is conductive and mechanically strong in both dry and wet conditions.

The addition of glycerol to the PEDOT-PSS silk string enhances the conductance and water resistance, which are advantageous properties for biomedical electrodes.

PEDOT-PSS, silk and glycerol combine well to form a physically stable hybrid material with high conductivity, good mechanical strength and tolerance to stress in both wet and dry conditions.

Also, utilizing the flexibility of its threads, we fabricated skin surface electrodes. ECG, electroencephalography (EEG) and sensory evoked potential (SEP) measurements were successfully performed on experimental animals.

## Results

### Features of fabricated PEDOT-PSS silk thread

**Figure 1:**
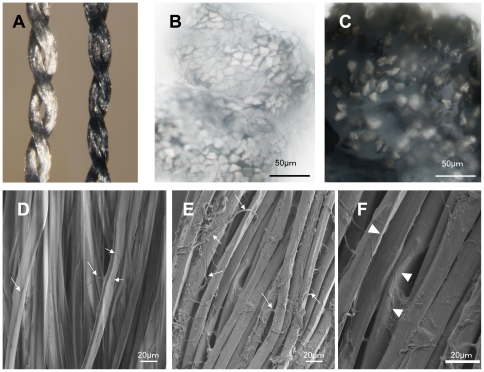
PEDOT-PSS silk thread observation with a conventional microscope and a scanning electron microscope (SEM). A. Macroscopic image of pristine silk thread (left) and PEDOT-PSS combined silk thread (right). B. C. Cross-section of pristine silk (B) and PEDOT-PSS silk thread (C) observed with a transmitted light optical microscope (scale bar 50 µm). D–F. Scanning electron micrograph of pristine silk (D) and PEDOT-PSS silk thread (E, Higher magnification view F). Arrows indicate fine filaments of silk fiber (D) coated with PEDOT-PSS (E). Arrowheads indicate PEDOT-PSS webbing between silk fibers. (F, Scale bar 20 µm). Pristine silk shows charge up and has a blurred image because of its insulation properties (D).

A PEDOT-PSS solution, mixed with 0.1% EDOT, was infiltrated into silk thread from *Bombyx mori* and electrochemically fixed [17]. Macroscopic images showed that each of the PEDOT-PSS silk bundled fibers were closely attached and formed a tighter bundle of fibers than pristine silk thread, suggesting that PEDOT-PSS causes silk fibers to adhere to each other (Fig. 1A). A membranous covering of PEDOT-PSS over the entire surface of the silk fibers was revealed by scanning electron microscope (SEM) observation (Fig. 1E, 1F). Also, a membrane webbing was observed in the gaps between the silk fibers indicating that PEDOT-PSS was deposited at the interspaces and on the surface of the silk thread.

### Distribution of PEDOT-PSS in silk thread

A transmitted light observation of a cross-section of the PEDOT-PSS silk thread showed that PEDOT-PSS was located between the silk fibers and the outside of the thread (Fig. 1C), and formed a 1∼10-µm layer. Some of the silk fibers appeared bright as a result of the transmitted light illumination, suggesting that they were not thoroughly stained by the PEDOT-PSS.

### Electrical property of PEDOT-PSS silk thread

The electrical conductivity of PEDOT-PSS silk thread was measured with a galvanometer at a constant voltage of 1 V (Table 1). The resistance of the PEDOT-PSS silk string, which consisted of a 280-µm diameter twisted silk thread made up of eighteen 21-denier silk fibers, was 9.04±1.45 MΩ/cm (average ± standard deviation number of samples = 10) and the conductivity was 1.84×10^−4^±2.81×10^−5^ S/cm (Table 1). Repetition of the electrochemical fixation, which increased the PEDOT-PSS content of the thread, improved its conductance. After a double fixation process, the resistance and conductivity were improved to 1.59±0.837 MΩ/cm and 1.35×10^−3^±8.08×10^−4^ S/cm, respectively (Table 1).

**Table 1:** Electrical conductivity of PEDOT-PSS silk thread; comparison of fixation and chemical additives.

PEDOT-PSS Fixation	Chemical Additive	Resistance (MΩ/cm)	Electrical Conductivity (S/cm)	n
single	-	9.04±1.45	1.84E-04±2.81E-05	10
single	Glycerol	0.0575±0.0173	3.04E-02±7.87E-03	23
double	-	1.59±0.837	1.35E-03±8.08E-04	13
double	Glycerol	0.0206±0.0124	1.02E-01±4.77 E-02	21
double	Ethylene Glycol	0.021±0.0150	9.63E-02±3.54 E-02	12
double	PPP PEG Copolymer 2000	0.0863±0.0314	2.18E-02±1.02E-02	21
double	PPP PEG Copolymer 2700	0.160±0.0966	1.59 E-02±1.21E-02	12
double	PPP PEG Copolymer 2500	0.102±0.0486	1.98E-02±1.01E-02	21

Average ± Standard Deviation.

### Mechanical property of pristine PEDOT-PSS and PEDOT-PSS silk thread in dry and wet conditions

**Figure 2:**
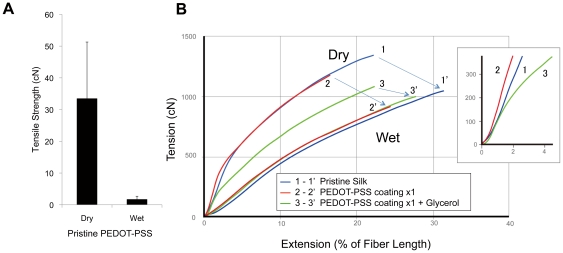
Mechanical properties of PEDOT-PSS silk thread. A. Tensile strength of pristine PEDOT-PSS film in dry (left) and wet (right) states (n = 10, error bar: 1 S.D.). B. Stress-elongation curves of pristine silk thread (blue lines 1, 1′), PEDOT-PSS silk thread (red lines 2, 2′), and glycerol treated PEDOT-PSS silk thread (green lines 3, 3′), in dry (1, 2, 3) and wet states (1′, 2′, 3′). The average data of 10 samples are plotted on the graph. Inset: Enlarged view of stress-elongation curves of the 3 groups in a dry condition in a lower strength range, pristine silk thread 1) PEDOT-PSS silk thread 2) and glycerol treated PEDOT-PSS silk thread 3). Glycerol treatment improved the elasticity of PEDOT-PSS silk thread.

PEDOT-PSS is hydrophilic and so water absorption greatly affects its mechanical property. The tensile strength of pristine PEDOT-PSS cast film (50 µm×1200 µm×2 cm) was 34.1±18.2 cN in a dry state, and fell to 1.71±1.05 cN (95% reduction) in wet conditions (Fig. 2A).

We measured the tensile strength of the fabricated PEDOT-PSS silk thread, which had almost the same cross-sectional area as the cast film. The thread exhibited a high tensile strength (1239 cN) that was almost equal to that of pristine silk (1350 cN) in a dry state (Fig. 2B). In a wet state, their tensile strengths were comparable to that of pristine silk (pristine silk 1083 cN and PEDOT-PSS composite 940 cN). Thus, the PEDOT-PSS was reinforced by the silk thread, which prevented the deterioration of the PEDOT-PSS by wetting.

### Glycerol improves the integration of PEDOT-PSS silk composite in wet conditions

**Figure 3:**
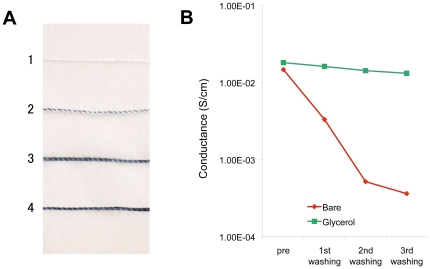
Glycerol enhanced water sturdiness of PEDOT-PSS silk thread. A. Photograph of the threads soaked in water for three days. 1) Pristine silk thread. 2) PEDOT-PSS silk thread without glycerol. 3) PEDOT-PSS silk thread with glycerol. 4) PEDOT-PSS silk thread without water soaking. B. Repeated cycles of washing with water and drying impaired the electrical conductivity of the PEDOT-PSS silk thread. The conductance of PEDOT-PSS silk thread (red line) and glycerol treated PEDOT-PSS silk thread (green line) after every washing cycle are shown (measured at dry state, averaged data from 10 samples are plotted). The conductance of thread that were not treated with glycerol fell to less than 1% that of the control. Glycerol treated thread retained its conductance after the third washing.

With water absorbed pristine PEDOT-PSS thread that exceeded the permissible water intake, there was separation of the PEDOT-PSS from the thread, which resulted in a loss of conductance. For example, when the PEDOT-PSS silk thread was immersed in distilled water (DW) for three days, the combined PEDOT-PSS was lost from the thread (Fig. 3A). To solve this problem, we tried to determine whether the addition of chemicals to regulate the wettability of the thread would increase its water resistance. We found that glycerol suppressed PEDOT-PSS degradation; the silk thread retained the blue-black color of PEDOT-PSS after being soaked for three days in DW (Fig. 3A). The water intake of the thread (0.101 g/cm weight increment after being soaked in DW from the dry state in RH50% at room temperature) was suppressed when glycerol was added (0.0218 g/cm, 78.4% reduction from control: without glycerol p<0.0001, n = 10). The water intake was suppressed by glycerol addition, and so PEDOT-PSS degradation may be attenuated, thereby increasing the water stability of a PEDOT-PSS-coated silk thread.

### Glycerol adds softness to PEDOT-PSS silk thread

Compared with pristine silk, PEDOT-PSS silk thread is rigid in dry conditions (Fig. 2B) due to the solidity of PEDOT-PSS. Thus, PEDOT-PSS silk thread feels coarse and stiff when attached to the skin. The Young's moduli (initial elastic moduli), calculated from initial part of the tensile elongation curves under dry conditions, of pristine silk, PEDOT-PSS silk and PEDOT-PSS silk glycerol were 2.41, 3.35 and 2.26 gPa, respectively (Fig. 2B inset). These data show that glycerol addition reduced the rigidity of the PEDOT-PSS silk thread and made the thread feel softer.

### Glycerol enhances the electrical conduction of PEDOT-PSS silk thread

It was reported that glycerol and other chemicals (polyethylene glycol, D-sorbitol and dimethyl sulfoxide DMSO) enhance the electrical conduction of PEDOT-PSS [18], [19]. The addition of glycerol significantly improved the conductivity of the PEDOT-PSS single fixed silk thread to 3.04×10^−2^±7.87×10^−3^ S/cm (57.5±17.3 kohm/cm) from untreated thread (1.84×10^−5^±2.81×10^−5^ S/cm, 9.04±1.47 MΩ/cm, p<0.0001, Table 1). Glycerol treated PEDOT-PSS double fixed silk thread showed the highest conduction 0.102±0.0477 S/cm, 20.6±12.4 kohm/cm (Table 1). Other chemical additives were examined, which were reported to have 1) high affinity to PEDOT-PSS 2) high viscosity and 3) the ability to enhance the electrical conductance of PEDOT-PSS (Table 1) [20]–[24]. Other chemicals in Table 1, poly(ethylene glycol-ran-propylene glycol)∼2500 (PEG-ran-PPG 2500), poly(propylene glycol)-block-poly(ethylene glycol)-block-poly(propylene glycol)∼2000 (PPG-block-PEG-block-PPG2000) and poly(propylene glycol)-block-poly(ethylene glycol)-block-poly(propylene glycol)∼2700 (PPG-block-PEG-block-PPG 2700) also exhibited an increase in conductance. Glycerol treatment resulted in PEDOT-PSS silk thread having the highest conductivity among the screened chemicals. Polyethylene glycol increased the conductance to almost the same degree as glycerol, but was rapidly diffused from the PEDOT-PSS thread in water and the effect was lost (data not shown). Hydrophobic additives, such as squalane, squalene or mineral oil were not thoroughly infiltrated into the PEDOT-PSS, and were completely separated from the polymer in the aqueous environment, and so did not provide any improvement in water resistance or conductance.

### Glycerol maintained conductance of the thread in washing cycles

Stresses, such as repeated washing and drying cycles cause the PEDOT-PSS to separate from the silk thread thus degrading its electrical conductance (Fig. 3B red line). For biomedical interface applications, it is desirable to enhance the durability of the thread to wetting and drying cycles. Glycerol-treated PEDOT-PSS-coated silk threads maintained their conductance after three washing and drying cycles (Fig. 3B green line), suggesting that wettability control using chemical additives keeps the polymer integrated in the thread, thus ensuring that conductance is maintained after the washing cycles.

### Electrocardiograph (ECG) recording using PEDOT-PSS silk thread

**Figure 4:**
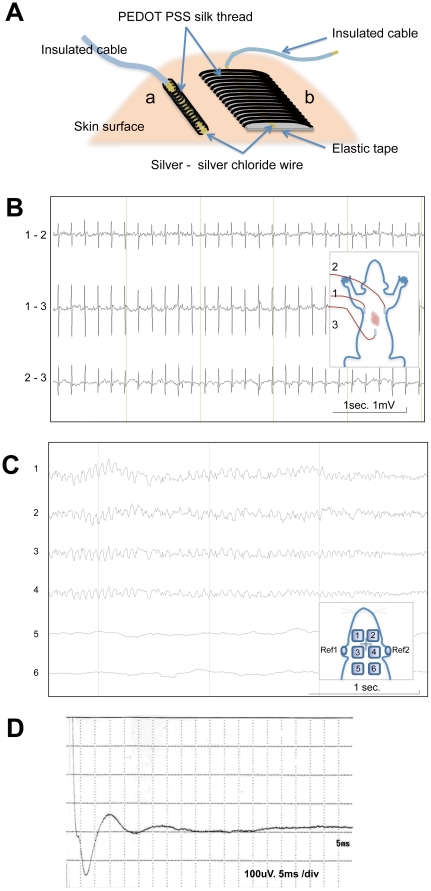
Electrophysiological recordings obtained using electrodes made with PEDOT-PSS silk glycerol thread. A. Structure of skin surface electrodes using PEDOT-PSS glycerol silk thread. a. String shaped electrode for electrocardiograph (ECG). b. Flat electrode for electroencephalogram (EEG). B. ECG recordings obtained from an experimental rat with bipolar deviation. Inset shows the position of the three electrodes. C. EEG recorded from a rat in an anesthetized condition. Inset shows the position of six different electrodes (flat PEDOT-PSS silk glycerol electrode, Fig. 4A, image b) on scalp and reference electrodes (Ag AgCl) on bilateral auricle. D. Sensory evoked potential (SEP) recorded from a rat parietal area with a flat PEDOT-PSS silk glycerol electrode induced by left posterior leg stimulations via (string shape PEDOT-PSS silk glycerol electrode, Fig. 4A, image a). Averaged data for 200 recorded signals are shown.

**Figure 5:**
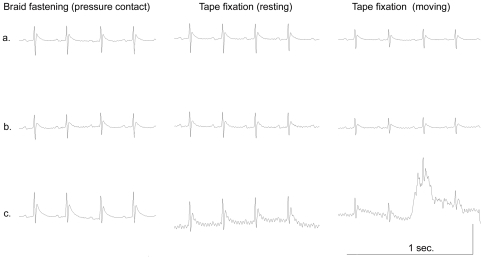
Comparison of ECG signals recorded from conventional, PEDOT-PSS silk thread and textile electrodes. Simultaneously recorded rat ECG signals from conventional medical electrodes (a), PEDOT-PSS silk glycerol thread electrodes (b), textile electrodes (c) pressure contact with skin by elastic band wrapping (pressure contact, left) or medical adhesive tape fixation without body movement (middle) and with body movement (right) are shown. ECGs recorded with PEDOT-PSS silk glycerol thread electrodes are comparable to ECGs obtained with conventional medical electrodes (a,b) under all conditions. The tape fixed textile electrode produced a hum (c, middle) and baseline drift (c, right) without and with body movement, respectively.

We used PEDOT-PSS silk glycerol thread to make a skin surface electrode with which to obtain biosignal recordings from the body. As shown in Fig. 4A, the electroconductive thread was coiled around fine silver wire, and then placed on the skin to conduct electrical signals to a cable connected to a bio signal amplifier. The electrodes were used to record electrical signals from rats. For ECG recording, three electrodes were placed on the anterior chest surrounding the heart to conduct myocardial signals. Figure 4B shows a representative ECG recorded from an anesthetized rat using PEDOT-PSS silk glycerol electrodes without employing gel or paste electrolytes. The quality of the recorded data was comparable to those obtained with conventional metal electrodes with gel or paste (Fig. 5). Silver sliver chloride wire alone, without PEDOT-PSS silk thread being wound around the wire, showed instability of recording signals (hum noise and baseline drift), which resembled the recording signal from silver yarn stitched textile electrodes (data not shown).

### Electroencephalogram (EEG) recordings using PEDOT-PSS silk electrodes

To record brain signals, flat PEDOT-PSS silk glycerol electrodes (7×12 mm, as shown by b in Fig. 4A) were placed on shaved skin and covered with an elastic braid to fit them to the scalp. The different electrodes were placed bilaterally on the frontal, parietal and occipital areas of the scalp. Reference electrodes were placed in bilateral ear auricles as shown in the Figure 4C inset. Figure 4C shows EEG signals recorded from a rat brain via 6 different electrodes under isoflurane anesthesia.

### Sensory evoked potential (SEP) recording using PEDOT-PSS silk electrodes

The PEDOT-PSS silk glycerol electrodes were examined for application to electrical stimulation. The stimulation electrodes (4×20 mm) were placed on the lower leg of a rat to stimulate the skeletal muscles. The gastrocnemius muscle was contracted by the stimulation (0.2 msec 6.4 mA square pulses interval 0.5 sec) through the PEDOT-PSS silk glycerol electrodes to the same extent as when using a conventional metal electrode with electrolyte paste. The evoked potentials are shown in Figure 4D by averaging the 200 signals recorded from the same electrodes used for EEG recording placed on the cortex. This result reveals that PEDOT-PSS silk glycerol electrodes are available for both stimulation and signal recording.

### Comparison of combined resistance with skin of PEDOT-PSS silk glycerol thread and conventional electrodes

Using experimental animals (rats), we measured the combined resistance of electrodes with skin at 10 Hz sine waves (RMS 1 v). As for the comparative data shown in Table 2, the impedance of the PEDOT-PSS silk glycerol electrode (0.2 kohm) was lower than that of conventional medical electrodes, Ag/AgCl with adhesive gel (32.3∼33 kohm) and a carbon electrode with a gel pad (35.4∼36.5 kohm).

**Table 2:** Combined resistance of conventional, PEDOT-PSS silk glycerol thread and textile electrodes on rat skin.

Electrode Type			Impedance (k ohm)	
	Dimension (mm)	Wet/Dry Condition	Tape fix	Elastic braid fix
Ag AgCl with adhesive gel for ECG	18×35		33	32.3
Carbon with gel & adhesion pad for ECG	Φ20		36.5	35.4
Ag AgCl with NaCl immersed sponge for EEG	Φ10	wet	0.1	<0.1*
PEDOT-PSS silk glycerol thread	7×12		0.2	0.3
Silver plated yarn stitched textile for ECG	25×40	dry	>200*	>200*
		wet	37.3	115
Silver plated yarn woven textile for sports	20×30	dry	>200*	>200*
		wet	-**	-**

* Outside measurement range.

** Impossible to measure because of electrode depolarization.

Textile electrodes, namely a silver yarn stitched elastic band [25] and silver yarn woven textile [11], both showed higher resistance than conventional Ag/AgCl gel electrodes when not soaked with water. The combined resistance of the textile electrode was higher than the measurement limit (200 kohm) of the electrode impedance meter. When the textile electrode was soaked with water, the composite resistance of the silver yarn stitched elastic band decreased to 1.67 kohm. The composite resistance of the silver yarn woven textile electrodes in a wet condition could not be measured owing to the electrode polarization.

The combined impedance of the textile electrode was changed when the contact condition with the skin changed. When the textile electrode was fixed in place with adhesive tape, it often exhibited impedance instability. The combined impedance was stabilized by wrapping an elastic band around the electrode to bring it into close contact with the skin.

The PEDOT-PSS silk glycerol electrode exhibited stable impedance when fixed in place with adhesive tape.

### Comparison of ECG signals recorded from conventional, PEDOT-PSS silk thread and textile electrodes

Signals recorded using PEDOT-PSS silk thread were compared with those obtained using conventional electrodes. ECGs were simultaneously recorded from a rat with three types of electrodes, namely conventional Ag/AgCl with adhesive gel electrodes (Vitrode P, Nihon Koden, Japan), PEDOT-PSS silk glycerol thread electrodes, and textile electrodes consisting of silver plated nylon yarn stitched to an elastic band [25] (Fig. 5). When the electrodes were brought into contact with the skin under pressure by fastening the elastic braid over them, the recorded signals obtained from all three electrodes were similar. Without using elastic braid pressure contact, adhesive tape fixation or self adhesion with a gel pad, the recorded signals from the e-textile electrodes frequently exhibited an increased hum noise and baseline drift owing to the change in the contact condition between the silver-plated yarn and the skin, which changed as a result of respiratory movement of the thorax (Fig. 5C). Conventional Ag/AgCl electrodes recorded the most stable ECG signals under all conditions. Signals recorded from PEDOT-PSS silk glycerol thread electrodes were stable against the respiratory movement of the thorax, which was similar to the result obtained with conventional Ag/AgCl electrodes (Fig. 5A, 5B). Without the addition of glycerol, the recorded signals obtained with bare PEDOT-PSS silk thread were as unstable as those obtained with textile electrodes without any pressure contact exerted by the elastic braid (data not shown).

## Discussion

The fabricated electroconductive thread, comprising a natural silk fiber bundle with PEDOT-PSS, provides a flexible, hydrophilic, electroconductive material that has been proved suitable for recording electrophysiological signals. Also, the addition of glycerol to the thread enhances durability in wet conditions, as well as flexibility and electro conductance. By using electrodes made of this material, electrophysiological signals, namely ECG, EEG, and SEP, were successfully transmitted from the surface of the body through metal cables. Electrical stimulation is also available through a thread placed on the skin.

Our prescreening of fiber affinity to PEDOT-PSS, using cotton, wool, nylon, polyester, cellulose and silk fibers, showed that silk has the best affinity to PEDOT-PSS especially in wet conditions (data not shown). Silk fibers consist of fibroin, a protein that has both hydrophobic and hydrophilic sites and that can absorb water in the fiber. SEM and conventional microscope observations showed that PEDOT-PSS adheres seamlessly to the silk fibers and forms an integrated bundle. Also, the fine filaments that split from the silk fibers entangled with the PEDOT-PSS and neighboring fibers, and this is thought to increase the adhesive strength between silk and PEDOT-PSS, thus stabilizing the composite structure.

In our study, silk thread was used as a partner of PEDOT-PSS for electrode fabrication, instead of a silk sheet. Although a flat sheet is geometrically more efficient than thread for conducting electrical signals from a flat skin surface, we noticed that silk thread offers advantages as regards electrode fabrication and utility compared with a silk sheet. Firstly, it was difficult to polymerize PEDOT-PSS uniformly on a silk sheet because the electrical current could not be controlled uniformly on the irregular surface. We found that the electrical polymerization of PEDOT-PSS in silk thread was technically easier than polymerization on a silk sheet, because, as with a metal cable, the current density of PEDOT-PSS soaked silk thread can be simply controlled with a regulated power supply. Secondly, electroconductive threads have the potential to expand the utility of biomedical electrodes. Thread can be stitched to clothes or woven into a strap to fabricate textile electrodes for electrophysiological testing, biomedical monitoring or ubiquitous health care systems.

In our study, we surveyed chemical additives to improve the functionality of PEDOT-PSS silk thread. Glycerol improved both the electrical property and water resistance of the threads in our screening. PEDOT-PSS is a rigid polymer in a dry state that is changed into a soft hydro-gel by water absorption. Once saturated with water, PEDOT-PSS breaks and peels from the thread resulting in reduced electric conduction. The addition of glycerol to PEDOT-PSS silk thread effectively stabilized the PEDOT-PSS in the thread in a wet state.

Glycerol addition provides the highest electroconduction characteristic in our system. It has been reported that glycerol and other chemical additives (e.g. D-sorbitol, polyethylene glycol and DMSO) increase the conductance of a PEDOT-PSS thin layer, cast film and microfibers.

Glycerol enhanced both PEDOT-PSS conductance and string flexibility thus improving adherence to the skin surface; this reduced the electrode and contact impedance, respectively. The signal to noise ratio was expected to improve. A comparison of glycerol treated and untreated electrodes showed that the background noise level was suppressed to less than 1/20 its original value by glycerol addition in our recording system, which is beneficial when recording weak signals e.g. EEG, SEP. Glycerol treatment is expected to protect PEDOT-PSS from wetting by sweat or body fluid and to keep its electro conductance in a practical range. Glycerol also improves the flexibility of PEDOT-PSS silk thread, and the way it feels, which is important for skin surface electrode applications, especially for long-term bioelectric recording, e.g. Holter ECG, and Holter EEG.

Our fabrication method delivers a composite consisting of PEDOT-PSS and silk thread where the PEDOT-PSS is distributed between the silk fibers and around the thread (fiber bundles). A cross-sectional view of PEDOT-PSS silk fibers shows that most of the PEDOT-PSS adheres to the outside of the silk fibers. This polymer distribution is believed to affect the electrical property of this fiber. The electrical conduction of the fabricated PEDOT-PSS silk fiber was much lower than that of previously reported pristine PEDOT-PSS cast film (0.8 S/cm) [26]. The inside of the silk fibers was not thoroughly stained by PEDOT-PSS, suggesting that silk fibers basically act as an electrical insulator that reduces the conductance of the thread. The resistance is improved by increasing the PEDOT-PSS content of the thread and also by the addition of glycerol. Additionally, a new electrode design and a high input impedance preamplifier placed at the recording site make it possible to collect low voltage signals such as EEGs.

Recently, biomedical textile electrodes have been developed for sports, ubiquitous healthcare and medical applications. Electrical measurements performed with PEDOT-PSS silk glycerol thread electrodes, textile electrodes and conventional medical electrodes have shown that PEDOT-PSS silk glycerol thread electrodes exhibit the lowest combined resistance with skin. Textile electrodes showed a higher resistance than conventional medical electrodes and PEDOT-PSS silk glycerol thread electrodes. The resistance of textile electrodes is closely related to the total area of silver plated yarn attached to the skin (data not shown). Thus, an expansion of the effective area of textile electrodes or an increase in the number of silver yarn stitches (woven) on the textile reduces the resistance. Conventional Ag/AgCl medical electrodes, which are equipped with adhesive gel pads, exhibited the most stable signals, indicating that an adhesive gel pad effectively stabilizes the conductive conditions of electrodes to skin during body movements. Textile electrodes needed to be tightened with elastic braid to stabilize ECG recording, which indicated that the contact condition of textile electrodes with the skin was more sensitive to body movements than conventional ECG electrodes.

Despite their structural similarity, PEDOT-PSS silk glycerol threads showed stable signals during thorax movements. Glycerol addition enhances the softness of PEDOT-PSS silk threads, and recordings using PEDOT-PSS silk thread electrodes without glycerol became unstable due to movement, which suggests that the contact of the PEDOT-PSS silk thread with the skin was improved by glycerol via the improved flexibility of the threads.

PEDOT-PSS silk glycerol electrodes were used as stimulation electrodes to induce sensory evoked potentials in rat brain. In this case, posterior limb muscles were successively contracted by square pulses of low current (0.2 msec 5–7 mA) injected through the PEDOT-PSS silk glycerol electrodes, owing to the small size of the animal body. However, further experiments are required to confirm that this new electrode is available for larger current density stimulation for large animal or human bodies.

To test the long-term usage of PEDOT-PSS silk glycerol threads electrodes were continuously attached to rats whose ECGs were recorded daily without changing the electrode. PEDOT-PSS silk glycerol threads electrodes were able to record ECGs for three days after attaching the electrode (data not shown). In this experiment using rats, it was hard to test for more than three days without replacing the electrodes because the rats' hair grew and detached the electrodes from their skin. For long-term usage, PEDOT-PSS silk glycerol threads electrodes must be fixed securely to the skin. The insecure attachment of the PEDOT-PSS silk electrode gradually increased electrode resistance, because the electrode slipped repeatedly on the skin thus removing the PEDOT-PSS coating from the silk threads. We believe that this electrode could be used as a disposable device for a few days if attached securely to the skin.

PEDOT-PSS silk thread is composed of hydrophilic materials that absorb water and electrolytes and so could provide an ideal interface between metal electrodes and living tissues. Further studies are required to confirm its biocompatibility including immune reactions, skin irritation, PEDOT-PSS metabolism and a further improvement of durability for long-term monitoring or permanent implantation.

We described a PEDOT-PSS silk glycerol thread that has flexibility, controlled hydrophilicity, scalability of size, reliability in wet conditions and biocompatibility. For biomedical and health information technology, PEDOT-PSS silk glycerol thread offers a new class of biocompatible electrodes that do not induce stress in the subjects.

## Materials and Methods

### Fabrication of PEDOT PSS combined silk thread

*Bombyx mori* silk thread (Fujix Japan) composed of a twisted bundle of eighteen 21-denier silk fibers was immersed in PEDOT-PSS (Clevios P Heraeus Germany) plus 0.1% v/w EDOT (Heraeus Germany) mixed solution for three days at room temperature. The silk string ran through rollers to remove any PEDOT-PSS polymer aggregates. A voltage was applied to the PEDOT-PSS 0.1% EDOT soaked silk strings by using stainless steel array electrodes connected to a DC power supply with a current regulator (PAB18-5.5 Kikusui Denshi Japan), which was in turn connected to a multimeter (VOAC7511 Iwatssu Japan) to monitor the electrical current. The applied voltage of 0.8∼18 V and string running speed (0.1∼10 cm/sec) were depended on the diameter of the thread and the desired amount of PEDOT-PSS fixed in the thread. After the electrical fixation, the thread was immersed in 100% ethanol for 30 minutes to remove unfixed PEDOT-PSS and EDOT, and then dried with an air blower. Glycerol, poly(ethylene glycol-ran-propylene glycol)∼2500 (PEG-ran-PPG 2500), poly(propylene glycol)-block-poly(ethylene glycol)-block-poly(propylene glycol)∼2000 (PPG-block-PEG-block-PPG2000), poly(propylene glycol)-block-poly(ethylene glycol)-block-poly(propylene glycol)∼2700 (PPG-block-PEG-block-PPG 2700) were purchased from Sigma (Sigma-Aldrich Japan). The electrochemically fixated and dried threads were immersed in each solution for more than two days at room temperature. Excess solution was absorbed by a paper towel before the thread was used.

### Electrical conductivity and tensile strength measurements of the threads

The electrical conductivity of the PEDOT-PSS combined silk threads was measured along a distance of 1 cm with two miniature test clips (Nano Clip, Stack Electronics Japan). 1.0 V DC was applied to the strings with a power supply (PAB18-5.5 Kikusui Denshi Japan), and two-point electrical conductivity measurements were performed with a multimeter (VOAC7511 Iwatsu Japan). Stress elongation curves were measured according to the Japanese Industrial Standard 1013 (thread length 20 cm, traction speed 20 cm/min). The average data for 10 samples were plotted on a graph.

### Evaluation of water resistance and absorption of PEDOT-PSS silk thread

The PEDOT-PSS silk threads (10 cm, with and without glycerol addition) were placed in a 12-well plastic dish (Falcon) filled with distilled water (DW, 20∼24°C) and shaken (60 rpm, tilt angle 5°) for 15 minutes on a rocking shaker (Wavemixer WEB-301 ASONE Japan). The threads were then dried for 24 hours at room temperature (24°C RH50%) and their electrical conductivity was measured in dry state. The washing and drying cycle was repeated three times. Water absorption was examined by increasing the weight of the 10 cm thread by soaking in it DW from the weight of the thread that was saturated with moisture at 24°C RH50%, and measuring the result with a precision balance (XP26 Mettler Toledo).

### Optical microscopy

The electrochemically fixed PEDOT-PSS silk thread was embedded in transparent epoxiresin (Epoclear Konishi Japan) for sectioning. To observe the PEDOT-PSS distribution in silk fibers, a sample string was transversely sectioned with a microtome blade (CTDMIC BLADES Leica Germany) at 500–1000 µm and observed with an optical microscope (BX51 Olympus Japan).

### Scanning Electron Microscopy

The surface microstructures of the fibers were characterized using a scanning electron microscope (Ultra 55 Zeiss Germany) with an applied voltage of 1 kV. PEDOT-PSS silk fibers were sufficiently conductive to be observed with SEM without a gold coating.

### Signal recording from experimental animals using PEDOT-PSS silk electrodes

The animal experiments were performed according to the guidelines for animal experimentation provided by the Ministry of Education, Culture, Sports, Science and Technology in Japan (Notification No. 71, 2007) and with the agreement of the Animal Ethics Committee of NTT Basic Research Laboratory (approval ID2011-01). Sprague-Dawley rats (Charles River Japan) were anesthetized with a gas mixture of 1–3% isoflurane and air during preparation and signal recording. Every effort was made to minimize suffering. The body temperature was kept at 37°C by using a warming pad designed for use during animal surgery. The skin hair at the recording area was shaved with a razor. The area was then sprayed with 83% ethanol (body and limbs) or 0.1% chlorhexidine gluconate (head and neck) for sterilization. All the electrodes were attached after the shaving and stylization without any additional preparation such as the removal of the superficial epithelial corneum or the application of electroconductive paste.

### Skin electrode combined impedance measurements

Target electrodes were placed on the shaved skin of the rat. As a reference electrode, we used a Ag/AgCl electrode (NE-134A, Nikon Koden Japan) with a cotton sponge that had been immersed in saline (0.9%NaCl) placed between the Ag/AgCl electrode and skin. The reference electrode was placed 4 cm from the target electrodes. The combined impedance of the electrodes and the skin was measured with a bio-electrode impedance meter (10 Hz RMS 1 v sine waves, Bioelectrode impedance meter, Melon Technos, Japan). Textile ECG electrodes designed for sports use were purchased (Smart fabric sensor, Polar, Finland). Textile ECG electrodes using silver yarn were fabricated according to reference [25]. Briefly, the silver plated nylon yarn (117/12×2 ply Shieldex Germany) was stitched using zigzag lines to a 2×10 cm elastic band (Spandex) by sewing machine.

### Fabrication of PEDOT-PSS silk glycerol threads electrodes

Two types of electrodes, string electrodes (for ECG recording) and flat shape electrodes (for EEG recording), were fabricated using PEDOT-PSS silk glycerol threads. String shaped ECG electrodes were made from PEDOT-PSS silk glycerol threads coiled around a fine Ag AgCl wire bundle to form the string shaped electrode shown in Figure 4A, a. Ag/AgCl wire bundles were made from silver micro wire (0.05 or 0.1 mm silver wire, Oyaide Electro, Japan), which was electrochemically formed on a/AgCl surface by using 0.8–1 V DC in a 1 M NaCl solution. The Ag/AgCl wire bundle was completely enclosed by PEDOT-PSS silk glycerol threads. The electroconductive part of the electrode was 1 mm in diameter and 1.5 cm long. Silver micro wire was connected to standard copper cables from biophysical amplifiers with soldering or small size crimped terminals.

The flat electrodes for EEG recording have the same structure as the ECG electrode except for an internal cushion (polystyrene foam tape, 7×12×2 mm) and an expanded contact area of PEDOT-PSS silk glycerol thread on the skin designed to reduce the combined impedance of the electrode and the skin.

### ECG recording by PEDOT-PSS silk electrodes

Three PEDOT-PSS silk glycerol thread electrodes were placed on the anterior chest of an anesthetized rat as shown in the Figure 4B inset. The electrodes were placed on shaved skin and fixed with medical adhesive tape (Silkey Pore, Alcare Japan) without using gel or paste electrolytes. Ag/AgCl wire was connected to the cable from a bio signal recorder (AP1024 TEAC Japan) to record ECGs at time constant 0.03, (high pass filter 30 Hz, sampling 1000 Hz). To compare the ECG signals from three types of electrodes, the electrodes were placed as close as possible on both sides of the lateral chest wall and recordings were made simultaneously. The electrodes were kept in contact with the skin by using an elastic braid fastening (pressure contact) or medical adhesive tape fixation (Silkey Pore, Alcare Japan).

### Electroencephalogram (EEG) recordings

For EEG recording, the square PEDOT-PSS silk glycerol electrodes were placed on shaved skin and covered with elastic braid for fitting to the scalp. The Different electrodes were placed bilaterally on the frontal, parietal and occipital areas of the scalp as shown in the Figure 4C inset. For the reference electrodes, conventional dish type Ag/AgCl electrodes with a cotton sponge that had been immersed in 0.9% NaCl were placed in the bilateral ear auricles. All electrodes were connected to a bio signal recorder (AP1024 TEAC Japan) at 1000 Hz sampling. The EEGs were recorded in a Faraday cage to reduce hum.

### Sensory evoked potential (SEP) recording

Stimulation electrodes with a larger contact area (4×20 mm) than the ECG electrodes but the same construction (Figure 4A, a) were placed on the lower leg skin of the rat close to the gastrocnemius muscle in the hind limb. The PEDOT-PSS silk glycerol electrodes were placed on the parietal area of the scalp. 200 square pulses (duration 0.2 msec, intensity 6.4 mA, interval 0.5 sec) were used for the stimulation. The electrodes were directly connected to an evoked potential recording system (Neuropack MEB-5504 Nihon Koden Japan) and 200 signals were averaged.

### Statistical analysis

All statistical analysis was undertaken with a non-parametric Kolmogorov-Smirnov test in SPSS statistics (SPSS USA).
